# Quercetin-Loaded Polycaprolactone-Polyvinylpyrrolidone Electrospun Membranes for Health Application: Design, Characterization, Modeling and Cytotoxicity Studies

**DOI:** 10.3390/membranes13020242

**Published:** 2023-02-17

**Authors:** Gianluca Viscusi, Gaetana Paolella, Elena Lamberti, Ivana Caputo, Giuliana Gorrasi

**Affiliations:** 1 Department of Industrial Engineering, University of Salerno, Via Giovanni Paolo II, 132, 84084 Fisciano, Italy; 2 Department of Chemistry and Biology “A. Zambelli”, University of Salerno, Via Giovanni Paolo II, 132, 84084 Fisciano, Italy

**Keywords:** electrospinning, wound healing, nanofibers, cytotoxicity, quercetin

## Abstract

Fibrous membranes of polycaprolactone (PCL)-polyvinylpyrrolidone (PVP) encapsulating 15% wt of quercetin are fabricated by a uniaxial electrospinning technique. Morphological analysis of the electrospun systems proved the fabrication of micrometric fibers (1.58 µm for PCL/PVP and 2.34 µm for quercetin-loaded membrane). The liquid retention degree of the electrospun membranes is evaluated by testing four different liquid media. The contact angle estimation is performed by testing three liquids: phosphate buffer solution, basic solution (pH = 13) and acidic solution (pH = 3), showing high hydrophobicity degree (contact angles > 90°) in all cases. The release of quercetin from the nanofibers in PBS (phosphate buffer solution) and pH = 3 medium, modeled through different models, shows the possibility of a fine tuning of drug release (up to 7 days) for the produced materials. The release profiles attained a plateau regime after roughly 50 h up to 82% and 71% for PBS and pH = 3 media, respectively. Then, since quercetin is known to undergo photooxidation upon UV radiation, release tests after different UV treatment times are carried out and compared with the untreated membrane, demonstrating that the release of the active drug changes from 82% for no-irradiated sample up to 57% after 10 h of UV exposure. The biology activity of released quercetin is evaluated on two human cell lines. The reported results demonstrate the ability of the quercetin-loaded membranes to reduce cell viability of human cell lines in two different conditions: direct contact between cells and quercetin-loaded membranes and cells treatment with culture medium previously conditioned with quercetin-loaded membranes. Therefore, the reported preliminary data confirm the possibility of applying the electrospun quercetin-loaded PCL-PVP membranes for health applications.

## 1. Introduction

Electrospinning has been emerging in recent decades as an interesting technique for producing nano and micro polymeric fibers for targeted applications [1,2,3,4]. It represents an excellent and versatile technique for the fabrication of micro- and nano-fibers using almost any type of polymer, either synthetic or natural [1,2,5,6,7,8]. The electrospun fibers are known to be potentially used in different technological fields, such as wound healing, oil removal, gas shielding, drug delivery, protective clothing, sensors and tissue engineering [1,9,10,11]. Among all, electrospun systems appeared to be promising as scaffolds for biomedical uses as well as medical patches [12,13,14] given their interesting properties, such as high drug loading capacity, high surface area, mechanical strength, porosity and low cost [15,16,17,18]. These fibers have the ability to incorporate various therapeutic substances with several functionalities, such as bacteriostatic or bactericidal activities and the capability to accelerate the healing process of the wounds [19]. In modern medicine, novel tunable and controlled systems for faster healing of lesions need to be designed in order to allow higher efficiency and controllability, and to reduce the side effects when treating different diseases. Functional molecules as drugs can be encapsulated in electrospun fibers in order to tailor and control the release kinetics for a targeted application. In fact, some diseases are required to be treated by guaranteeing a controlled drug concentration over time, which may require to tailor the release rate of drug delivery [20]. Moreover, the encapsulation inside a nanometric fiber could provide further protection against the fast inactivation caused by patient body or environmental parameters. In this research, polycaprolactone and polyvinylpyrrolidone were selected as biomaterials for the targeted application, while quercetin was chosen as functional molecule. Polycaprolactone (PCL) is a Food and Drug Administration (FDA)-approved synthetic polymer which is biodegradable and can be dissolved in methanol, acetic acid, chloroform, benzene, trifluoroethanol and dichloromethane [9]. PCL can be easily incorporated with antibiotics, growth factors, bioactive compounds and proteins [21,22]. Although PCL is known to possess a good biocompatibility and efficacy both in vitro and in vivo, since the high hydrophobicity and the slow degradation kinetics, its applications in biomedicine are limited. Blending techniques have been widely used to modify physical and chemical properties of PCL. This methodology could improve the water diffusion to the proximities of PCL chains leading to the speeding up of the hydrolytic degradation [23]. In this framework, this work focused on the use of PCL blended with polyvinylpyrrolidone (PVP) since its good biocompatibility, solubility in water and organic solvents, and capability to interact with many hydrophilic and hydrophobic materials [24]. PVP deserves special attention due to its good environmental stability and easy processability [25]. It has been mainly used as an enhancer of the dissolution of pharmaceutical formulations. In addition, it can form freestanding films, fibers and hydrogels after certain modifications such as thermal treatment or UV-mediated radical reactions. The ability of PVP to incorporate and release antibiotics in a sustained manner and its self-adhesive properties have been recently demonstrated [26]. Both polymers, PCL and PVP, are known to show tissue-compatibility and no toxicity. Indeed, they are characterized by very different degradation times, and, therefore, the competition between diffusion and erosion mechanisms can help tailor the release depending on the necessity. Quercetin (C_15_H_10_O_7_-3,3′,4′,5,7-pentahydroxyfavone), a polyhydroxy compound and a natural flavonoid substance, has gained much attention in recent years due to its beneficial properties to human health. Quercetin is an acicular crystal, yellow and slightly bitter, and it has a number of bioactivities [27]. It is abundant in apples, onions, teas and red wines, where it is present as a glucoside [28]. It has been found to exert several biological activities such as antioxidant, antitumoral, antimicrobial and antiviral [29]. In addition, it has been reported to exert antidiabetic effect in vivo [30], to enhance wound healing in treated individuals [31] and to exhibit effects to prevent colonic carcinogenesis [32]. In nervous system tissue, quercetin has shown to limit apoptotic cell death, fibrosis and scar formation after injury, supporting its use as a model drug in the context of nerve repair and regeneration [33]. As a drawback, these therapeutic effects of quercetin is impeded due to its low water solubility, short half-life, light induced degradation, and rapid metabolism prior to reaching the systemic circulation [34]. In recent years, in order to improve its water solubility and stability, there have been many studies forming inclusion complexes with quercetin [35]. To overcome these problems, different delivery systems have been developed, and, thus, the use of quercetin with an appropriate carrier could improve its properties and minimize its degradation process [36]. In order to exercise its function, quercetin should be released at an appropriate rate. Therefore, in some cases, it is also desirable to release a large portion of the molecule. In order to avoid a too-fast and a too-slow release, a promising release behavior was obtained using a polymer blend system consisting of both hydrophilic and hydrophobic polymers. In this context, the scope of this article was to develop PCL-PVP blend fibrous scaffolds encapsulating quercetin as a biological active substance using electrospinning as a processing technique. The produced PVP-PCL nanofibrous systems have been widely characterized in order to prove the potential of the scaffolds as wound dressing materials for health applications.

## 2. Materials and Methods

### 2.1. Materials

Poly(ε-caprolactone) (PCL-average M_n_ = 80000-CAS: 24980-41-4) and polyvinylpyrrolidone (PVP-average M_n_ = 40000-CAS: 9003-39-8) were purchased from Sigma Aldrich. Chloroform (CHCl_3_-CAS: 67-66-3, 99.0–99.4%), methanol (CH_3_OH-CAS: 67-56-1, ≥99.8%), quercetin (Querc-CAS: 117-19-5, ≥95%), hydrochloric acid solution 37% *v*/*v* (CAS: 7647-01-0), urea (CAS: 57-13-6, 99.0–100.5%), lactic acid (CAS: 50-21-5, ≥85%), potassium chloride (CAS: 7447-40-7, 99.0–100.5%), sodium phosphate dibasic dihydrate (CAS:10028-24-7, ≥99.0%) and potassium phosphate monobasic (CAS: 7778-77-0, ≥99.0%) were purchased from Sigma Aldrich (Milan, Italy). Sodium chloride (CAS: 7647-14-5, 99.9–100.5%) was purchased from Carlo Erba Reagents (Cornaredo, Italy).

### 2.2. Fabrication of Quercetin-Loaded Membranes

The electrospun membranes were produced by dissolving PCL and PVP in a solvent mixture CHCl_3_/CH_3_OH (50:50 *v*/*v*) at 15% *w*/*w*. Quercetin was added to PCL-PVP (8:2 *w*/*w*) solution in order to guarantee a drug to polymer ratio of 15:85 (*w*/*w*) and mixed for 4 h at 40 °C (300 rpm) to obtain a homogenous solution. Before performing the experiment, the solutions were fed into a 5 mL syringe pump. The set of electrospinning conditions is optimized to produce bead-free fibrous membranes: flow rate = 0.7 mL/h, voltage = 18 kV, distance needle-collector = 21 cm. Temperature and relative humidity were fixed for all the experiments and equal to 25 °C and 35%. Climate controlled electrospinning apparatus (EC-CLI, IME Technologies, Geldrop, The Netherlands) was used to produce fibrous membranes, setting a vertical setup. The diameter of the needle was 0.8 mm. Finally, an aluminum collector was used to collect the electrospun nanofibers.

### 2.3. Methods

#### 2.3.1. Morphological Analysis

Scanning electron microscopy (SEM) analysis was performed using a Phenom ProX microscope in high-vacuum mode. Before the analysis, electrospun membranes were covered with a thin film of gold using an Agar Automatic Sputter Coater (Mod. B7341, Stansted, UK) at 40 mA for 120 s prior the analysis. Plot profiles were obtained from SEM images through Plot Profile plug-in of Fiji software. Image processing analysis allows the display of a two-dimensional graph of the intensities of pixels along the distance (pixel) by selecting an area of roughly 1000 μm^2^. The *x*-axis represents the distance along the line and the *y*-axis is the pixel intensity. Finally, surface roughness parameters were evaluated through mathematical equations. R_a_ (arithmetic average of the absolute values of the profile height deviations from the mean line, recorded within the evaluation length) and RMS (root mean square average of the profile height deviations from the mean line, recorded within the evaluation length (L)) were evaluated through Equations (1) and (2):

(1) $$R_{a}=\frac{1}{L}*\int_{0}^{L}\left|Z\left(x\right)\right|\mathrm{dx}$$

(2) $$\mathrm{RMS}={\left[\frac{1}{L}*\int_{0}^{L}Z{\left(x\right)}^{2}\mathrm{dx}\right]}^{\frac{1}{2}}$$

where Z(x) is the profile height function.

#### 2.3.2. Contact Angle and Liquid Retention Tests

Contact angle measurements were carried out by testing three different liquid systems: phosphate buffer solution (PBS, pH = 7.4), HCl solution (pH = 3) and NaOH solution (pH = 13). The tests were carried out by using a high-resolution camera at room temperature. Droplet of liquids (100 μL) were let spread onto the fibrous mat. The contact angle was evaluated using Drop Analysis software. Five tests were carried out for each sample.

Liquid retentions of electrospun fabric were performed by immersing a pre-weighed mass of sample (previously dried for 8 h under vacuum at 50 °C and weighed (M_0_)) in 25 mL of different liquid solutions (PBS, HCl solution (pH = 3), NaOH solution (pH = 13) and sweat simulant (pH = 5.5)). Sweat simulant was prepared according to EN 1811:2011 by dissolving NaCl (10.8 g), lactic acid (1.2 g), urea (1.3 g) in 1 L distillate water. pH was adjusted to 5.5 by dropping NaOH solution. Liquid retention tests were performed at 37 °C for 24 h. After that, the samples were re-weighed (M_eq_). The retention degree was evaluated according to the Equation (3):

(3) $$q\;\left(\%\frac{g}{g}\right)=\frac{M_{0}{-M}_{\mathrm{eq}}}{M_{0}}*100$$

#### 2.3.3. Release Kinetic of Quercetin

Release of quercetin was analyzed using a Spectrometer UV-2401 PC Shimadzu (Shimadzu Corporation, Kyoto, Japan). The tests were performed using rectangular specimens with an area of 2 cm^2^ in PBS solution (pH = 7.4) and HCl solution (pH = 3). The solution was stirred at 300 rpm in an orbital shaker (VDRL MOD. 711 + Asal S.r.l.). The release medium was withdrawn at set time intervals and replenished with fresh medium. The detection wavelength was set at 376 nm. The diffusion coefficients of quercetin from the fiber mat were also calculated using the following Equations (4) and (5) [37]:

(4) $$\mathrm{short}\;\mathrm{time}\;\frac{M_{t}}{M_{\infty }}=\frac{2}{L}\sqrt{\frac{\mathrm{Dt}}{\pi }}$$

(5) $$\mathrm{long}\;\mathrm{time}\;\frac{M_{\infty }-M_{t}}{M_{\infty }}=\frac{8}{\pi^{2}}\exp\left(-\frac{\pi^{2}\mathrm{Dt}}{4L^{2}}\right)$$

where D is the diffusion coefficient, L is the mat’s thickness.

#### 2.3.4. Photostability

In order to investigate the photostability of quercetin in PCL/PVP fibrous membranes, the samples were mounted 10 cm away from a UV lamp (150 W) and irradiated with UV-A light during pre-determined time intervals (from 30 min up to 10 h). After irradiation, both samples were immersed in 25 mL of PBS and the amount of released quercetin was evaluated after 24 h by UV–Vis spectroscopy. Each sample was analyzed in triplicate and the results are given comparative to quercetin released from non-irradiated systems.

### 2.4. Computational Work

Computation analysis and H interactions was performed using the Materials Studio 8.0 software by analyzing PCL-PVP network and Quercetin. The polymeric chains and the molecular system of quercetin were geometrically optimized using the Forcite module and COMPASS (Condensed Phase Optimized Molecular Potentials for Atomistic Simulation Studies) force fields. The polymers–drug system was analyzed through Amorphous Cell option while the interactions PCL-PVP were determined through Blends option. Three-dimensional cubic unit cells with periodic boundary conditions of 21.8 × 21.8 × 21.8 Å were constructed which comprised polymers and quercetin according to their weight ratio.

### 2.5. Biological Activity

#### 2.5.1. Cells Culture

To evaluate the biological activity of quercetin-loaded membranes, two human cell lines were employed: embryonal lung fibroblast cell line (MRC5), an adherent cell line, and acute myelogenous leukemia (KG1), a suspension cell line. Cell lines were obtained from Interlab Cell Line Collection (IST, Istituto Nazionale per la Ricerca sul Cancro, Genoa, Italy).

MRC5 was cultured in Dulbecco’s Modified Eagle medium (Gibco) supplemented with 10% (*v*/*v*) fetal bovine serum, 0.2 mM l-glutamine, 50 units/mL penicillin and 50 g/mL streptomycin. KG1 was culture in Iscove’s modified Dulbecco’s medium (Sigma Aldrich, Milan, Italy) supplemented with 20% (*v*/*v*) fetal bovine serum, 0.2 mM l-glutamine. All cells were grown at 37 °C, in a 5% CO_2_, 95% air-humidified atmosphere and passaged twice a week.

#### 2.5.2. Cell Viability Assay

Cell viability was evaluated by a 3-(4,5-dimethylthiazol-2-yl)-2,5-diphenyltetrazolium bromide (MTT) assay by two different experimental procedure: cells (KG1) were seeded in direct contact with 1 cm^2^ of quercetin-loaded membranes or cells (MRC5) were incubated with culture medium previously conditioned with quercetin containing films; in this case, 1 mL of medium was incubated in the presence of 1 cm^2^ of membrane at 37 °C for 24 h. As negative controls, non-loaded membranes or medium conditioned with non-loaded membranes were used. Membranes were sterilized by exposure to UV radiation for 1 h before used.

MTT assay was performed as previously described [38]. Briefly, MRC5 cells were plated at the density of 2 × 10^4^/cm^2^ in 96 wells plates 24 h before treatment, whereas KG1 cells were plated at density of 1 × 10^5^/cm^2^ in 24 wells plates 48 h before direct contact with quercetin loaded membranes. After the respective treatment, cells were incubated for 1 h with MTT and the resulting formazan crystals were dissolved in 100 μL of DMSO; absorbances were measured at 595 nm. The background signals at 655 nm were subtracted from 595 nm signals.

### 2.6. Statistical Analysis

Results were expressed as mean ± standard deviation (SD). One-way analysis of variance (ANOVA) and Tukey’s test were used for statistical comparison. For cell viability assays, Student’s t-test was used to calculate the statistical significance of differences between treated and untreated cells. Difference was regarded as statistically significant when *p* < 0.05. Coefficient of determination R^2^ was used to evaluate the goodness of the fitting processes.

## 3. Results

### 3.1. Morphological Analysis

**Figure 1:**
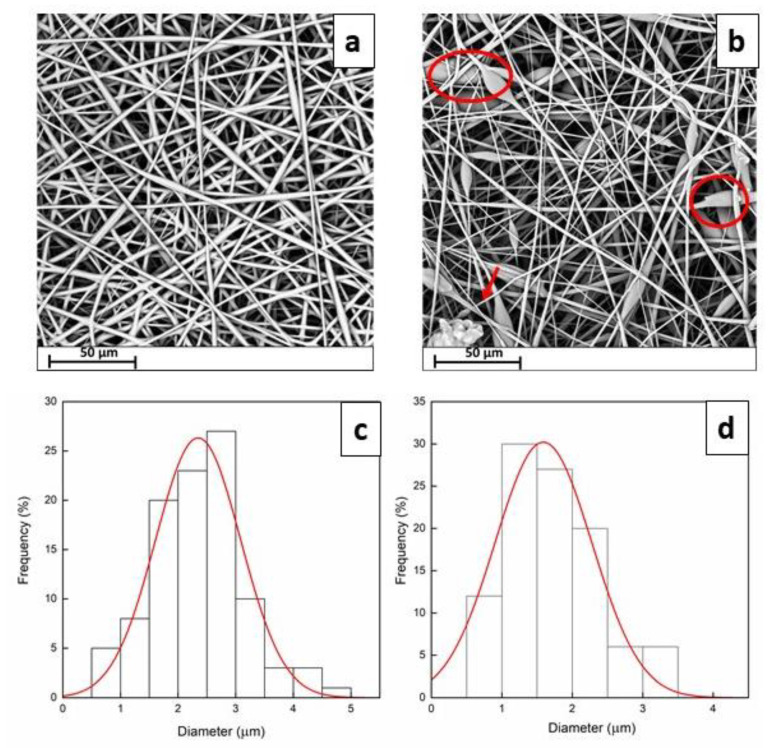
SEM images and diameter distributions of PCL/PVP (**a**) and Querc-PCL/PVP membranes (**b**). Distributions of diameters of PCL/PVP (**c**) and Querc-PCL/PVP (**d**). (red circles and arrows indicate the beads).

Electrospinning conditions were firstly optimized to produce defect-free fibrous membranes. By adopting the optimal conditions, fibers loaded with quercetin were successfully obtained. SEM images and the fiber diameter distributions of the electrospun membranes are shown in Figure 1.

**Figure 2:**
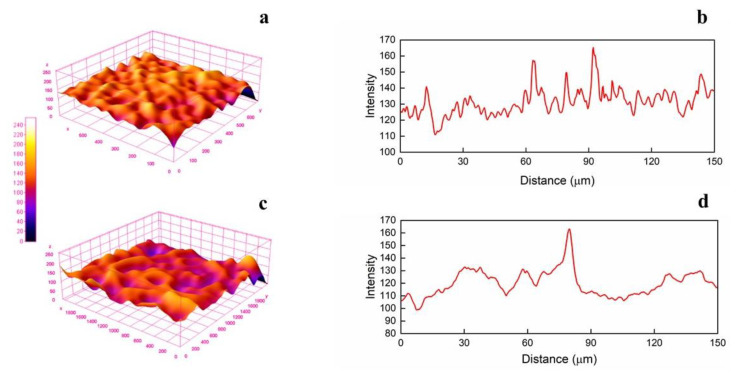
Three-dimensional surface plots of electrospun systems ((**a**): PCL/PVP and (**c**): Querc-PCL/PVP) and profile plots ((**b**): PCL/PVP and (**d**): Querc-PCL/PVP).

Both fibrous systems showed to possess a randomly fibers-oriented structure without defects. The average diameter is roughly 2.34 ± 0.21 µm for PCL/PVP and 1.58 ± 0.18 µm for Querc-PVL/PVP, respectively. This result indicates that, probably, addition of quercetin resulted in increased conductivity of the solution, which is known to affect the whipping motion of the electrospinning jet, thus leading to thinner fibers [28,39]. In fact, high electrical conductivity is supposed to have a downsizing effect on the diameters of fibers. The four OH groups and the benzene rings of quercetin could favor the charge transfer enabling to reduce the fiber diameter. The fibrous structure is fully formed with a small number of beads (marked by red circles), as shown in Figure 1. The presence of beads observed in the SEM image might be due to the presence of free quercetin on fibers surface as well as its agglomerates inside the fibers. The obtained structures do not possess a nanometric morphology, but, for the proposed application regarding the drug delivery, a smooth and bead-less nanofibers structure has a more significant effect than the fiber diameter [40]. By considering the SEM images, it could be stated that smooth and defect-free fibers were obtained, confirming that no phase separation occurred during the electrospinning process and suggesting a good dispersion of the quercetin molecules. The investigation of SEM micrographs even allowed to obtain the profile plots reported as gray value (pixel intensity) vs. pixel distance (Figure 2).

It is possible to observe a change in profile height deviations after the loading of quercetin. The gray intensity values of the systems increase up to 165 ± 17 and 163 ± 15 for PCL/PVP and Querc-PCL/PVP, respectively. In the three-dimensional surface profile, the topology clearly differs each other in height. Regions with very low pixel intensity values are seen as blue while regions with high intensity appeared to be red. The R_a_ values are 132 ± 12 and 117 ± 13 while the RMS are 132 ± 19 and 118 ± 10 for PCL/PVP and Querc-PCL/PVP, respectively. The 3-D maps in Figure 2 confirmed the trend of the parameters R_a_ and RMS.

### 3.2. Computational Analysis

**Figure 3:**
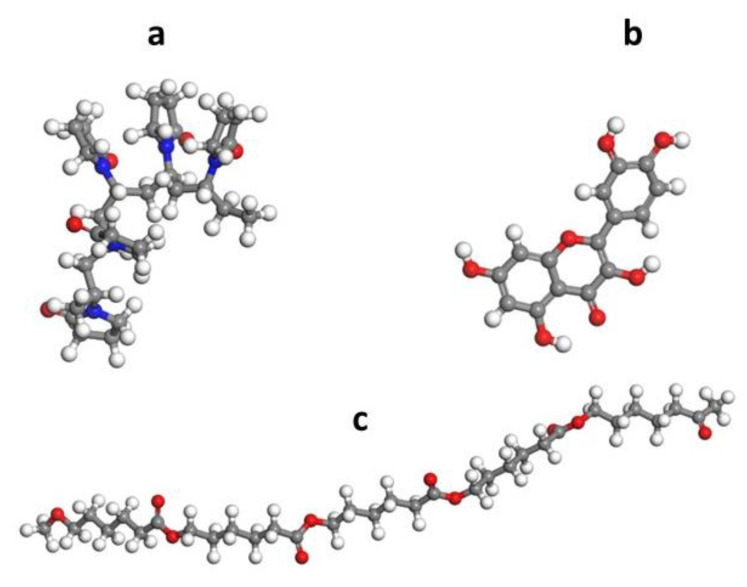
Optimized molecular structures of (**a**) PVP, (**b**) Quercetin and (**c**) PCL.

Both polymers and quercetin were constructed, and the geometrical optimization was carried out, through Forcite tool over 500 iterations, resulting in total energy minimization of the system: E_tot_ = 55.3 Kcal/mol for PVP, E_tot_ = 10.5 Kcal/mol for PCL and E_tot_ = 65.4 kcal/mol for quercetin. The geometrically optimized polymers chains and quercetin molecule are illustrated in Figure 3.

**Figure 4:**
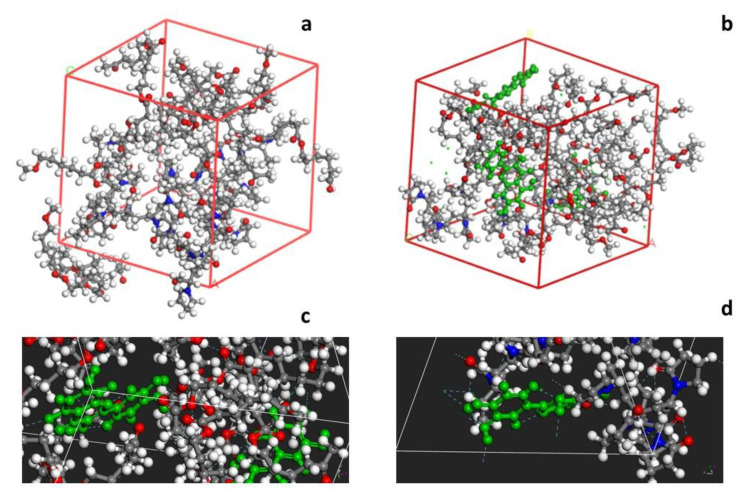
The amorphous unit cell of PCL/PVP (**a**) and PCL/PVP + Querc (**b**); H bond interactions between PCL and quercetin (**c**), and PVP and quercetin (**d**).

A three-dimensional cubic unit cell was constructed using the Amorphous module (Figure 4a,b). The optimized geometries of Querc-PCL/PVP exhibited hydrogen bonding interactions (cyan dotted line) between the OH group of quercetin (green molecule) and the C=O group of polymers as evident in 4c and 4s4d, which is confirmed by the Forcite module, with minimum total energy of E_tot_ = −913 kcal mol^−1^.

### 3.3. Liquid Retention Tests

**Figure 5:**
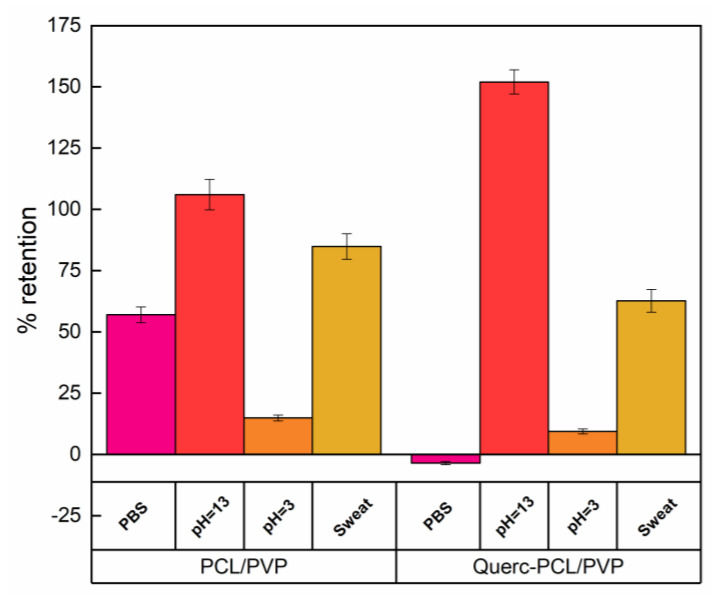
Liquid retention of electrospun membrane.

Figure 5 reports the liquid retention tests of pristine membrane and quercetin-loaded PCL/PVP fibrous systems.

The obtained data can only be described by considering different parameters affecting the retained liquid amount such as roughness, porosity, polar surface sites and isoelectric point. Concerning the test carried out by using phosphate buffer solution, an increase of 57% in weight was observed for PCL/PVP membrane, while a negative value, representative of weight loss, was recorded for the quercetin-loaded system. This trend can be correlated to the higher porosity of PCL/PVP systems allowing water to better go through fibers. Concerning the retention of sweat simulant, a reduction of 26% was observed. In this case, the overlapping of different factors has to be considered, such as porosity, ionic strength, isoelectric point and presence of salts. The presence of salts could lead to a partial deprotonation of quercetin; a negative charge onto the surface is present leading to the formation of weak electrostatic interactions with cationic groups. It follows that the swelling of polymeric system favored the entry of water molecules. Concerning acidic medium, data indicated a relatively stable material irrespective of quercetin loading. In acidic medium, retention degrees of 15% for PCL/PVP and 9% for Querc-PCP/PVP were observed. The slight decrease in retention for the loaded membrane could be due to the reduction in porosity caused by the presence of quercetin. This effect could be associated to the fact that, as pH < pk_a_, the neutral form of quercetin might exist and, so, the hindrance offered by quercetin molecules could limit the water diffusion inside the polymeric network. Concerning the alkali solution (pH = 13), an increase of about 43% was for Querc-PCL/PVP compared to the unloaded one. This could be due to the effect of pH on quercetin stability. The pk_a_ values for two subsequent deprotonations of OH groups of quercetin have been (pka_1_ ∼ 7.03 and pka_2_ ∼ 9.15) [41]. At about pH = 7, single deprotonated species of quercetin (i.e., monoanion) and neutral form of quercetin might coexist while as pH > pk_a_, the double deprotonated species of quercetin (i.e., dianion) might co-exist with another two (neutral and monoanion) species of quercetin. Therefore, at high pHs, the negative quercetin forms could exercise a repulsive force which led to the swelling of the polymeric structure allowing the water for entering into it.

**Figure 6:**
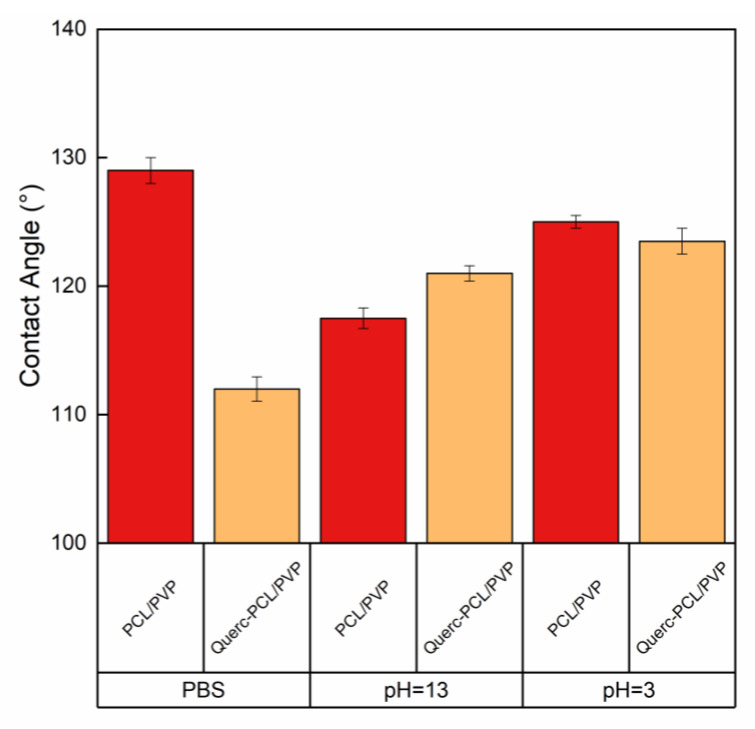
Contact angles values for PCL/PVP and Querc-PCL/PVP membranes.

The contact angles of the membranes were shown in Figure 6.

It is well known that the lower the contact angle, the higher the hydrophilic nature of the surface. The average PBS contact angle values were found to be 129° ± 1° and 112° ± 1° for PCL/PVP and Querc-PCL/PVP membranes, respectively. It is known that polyesters, such as PCL, are hydrophobic materials [42]. Therefore, this property could explain the high hydrophobicity of PCL/PVP membrane. The loaded membrane showed less hydrophobic character than the quercetin-unloaded membrane due to the presence of OH groups which could contribute to favor the spreading of liquid droplet onto the surface. In basic condition (pH = 13), the contact angle values were found to be 118° ± 1° and 121° ± 2° for PCL/PVP and Querc-PCL/PVP membranes, respectively, while in acidic condition (pH = 3), the contact angle values were found to be 125° ± 0.5° and 124° ± 0.6° for PCL/PVP and Querc-PCL/PVP membranes, respectively. Therefore, no noticeable statistical differences could be observed.

### 3.4. Release Kinetics of Quercetin

**Figure 7:**
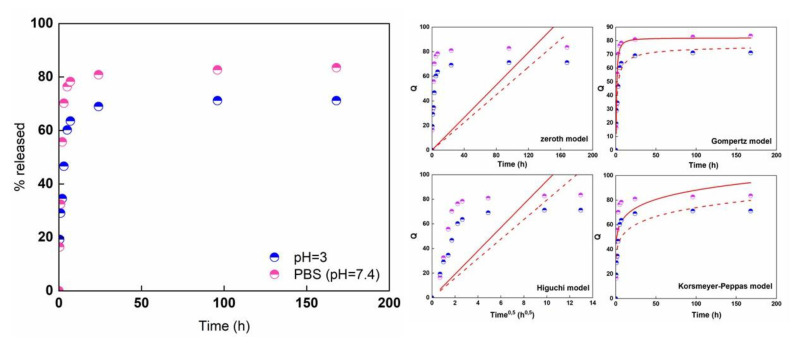
Release data of quercetin from electrospun systems in different media.

Since some wounds with pus or necrotic tissue are known to show an acidic pH [43], the release of quercetin was even tested in pH = 3 solution, apart from PBS (pH = 7.4). Figure 7 shows the release of quercetin in PBS and pH = 3 solution from pristine PCL/PVP and Querc-PCL/PVP membranes as function of time (h). The release profiles have complex behavior, characterized by a first release rapid step (burst), followed by a slower stage up attaining a plateau regime.

The initial burst can be mainly ascribed to surface desorption or pore diffusion. So far, a number of investigations strongly reported the limited release capability of quercetin into various media. Moreover, the release could even be affected by the polymer hydrophobicity as well as from the presence of intermolecular forces. In hydrophobic polymers, hydroxyl groups in their structure prevent miscibility of quercetin in the polymer and a tendency of quercetin molecules to cluster together. The presence of hydrophilic polymer enables the quercetin molecule to have comparatively high mobility [44]. Quercetin has both hydrophilic (OH groups) and rings as hydrophobic components. For a hydrophobic polymer, quercetin is not miscible in the polymer because its OH groups are exposed outside, and quercetin molecules also tend to cluster together, resulting in lower mobility. For a hydrophilic polymer, the OH groups make quercetin more miscible in the polymer, and the hydrophobic rings prevent quercetin molecules from binding to the polymer, both allowing quercetin to move more freely. Therefore, the presence of a polar polymer such as polyvinylpyrrolidone could aid the quercetin release. The burst release is more pronounced in PBS (78% after 7 h) compared to pH = 3 solution (63% after 7 h). In acidic conditions, since pH < pk_a_ of quercetin, the deprotonation did not occur. Therefore, neutral form of quercetin exists. As pH = 7.4 (PBS) > pk_a1_, single deprotonated species of quercetin is presents so, the partial charge located onto the surface, could favor the release from the fibrous system. This effect could explain the higher release amount in PBS compared to pH = 3. Then, the release profiles attained a plateau regime after roughly 50 h up to 82% and 71% for PBS and pH = 3 media. The release kinetics of drug from the loaded electrospun membrane can be studied by using zero-order (Equation (6)), Gompertz (Equation (7)), Higuchi (Equation (8)), and Korsmeyer–Peppas models (Equation (9)) [42,45]. The equations are listed below with Q denoting the fraction of total drug released up to time t and k as the kinetic constant of each model.

(6) $${Q=K}_{0}*t$$

(7) $${Q=Q}_{\max}*\exp[-\alpha *\exp(\beta *\log\left(t\right))]$$

(8) $${Q=K}_{H}{*t}^{0.5}$$

(9) $${Q=K}_{R}{*t}^{n}$$

Figure 7b shows the fittings of release data over the time range (solid line refers to the release evaluated in PBS while dot line refers to the release evaluated in acidic medium at pH = 3). Evaluated parameters are listed in Table 1.

**Figure 8:**
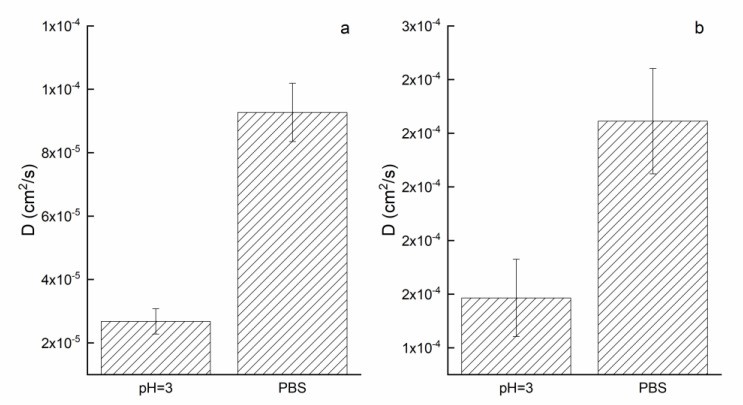
Diffusion coefficients (short (**a**) and long (**b**) times) of the electrospun systems in different media.

The trend suggests that release did not follow zero order kinetics, where quercetin would be released at a constant rate until a plateau regime was reached. It is possible that quercetin molecules furthest from the surface of the fiber may simply take longer to diffuse than quercetin molecules closer to the surface of the fiber. It is also possible that release is not solely directed by quercetin, indicating some degree of interaction between quercetin and the polymers. Moreover, the drug release, according to the Korsmeyer–Peppas model, was controlled via a typical Fickian diffusion mechanism (when *n* ≤ 0.5). Among the chosen models, a Gompertz equation seems to best fit the release data (high R^2^ coefficients). The Q_max_ is equal to 83% and 70% for PBS and pH = 3 solution while the parameter β is −2.7 and −1.5 for PBS and pH = 3 SLN. This latter parameter represents the dissolution rate per unit of time described as shape parameter. The diffusion coefficients (for short and long times) have been evaluated by analyzing the release profiles according to Equations (4) and (5) and reported in Figure 8.

As expected by observing the release profiles, the diffusion coefficients in PBS are higher than pH = 3 solution in both cases. In particular, the D (short time) in PBS is one order of magnitude higher than pH = 3 while, for long times, the D in PBS is 1.5 time higher than pH = 3. The mobility of quercetin molecules in slightly neutral solution (pH = 7.4) might be due to the presence of anionic quercetin which, since the generation of repulsive forces, is favored to be released faster compared to acidic medium.

### 3.5. Photostability

**Figure 9:**
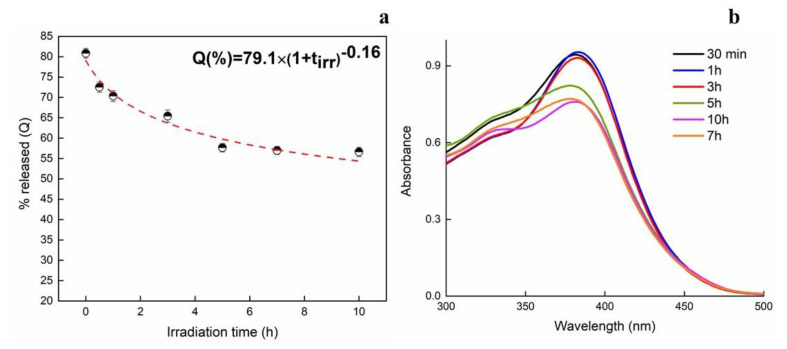
Release of quercetin after 24 h from irradiated samples (**a**) and UV-Vis spectra of quercetin at different irradiation times (**b**).

Since quercetin might undergo photooxidation upon UV irradiation, photostability is of vital importance for quercetin to show its activity [46]. In order to investigate the protective effect of microfibers on the photostability of quercetin, UV irradiation tests were performed at different time intervals. After irradiation, release tests were carried out in PBS after 24 h (Figure 9).

The release of quercetin from UV-irradiated samples is shown to decrease along irradiation time. It changes from 82% for no-irradiated sample up to 57% after 10 h of UV exposure. From the obtained data, it can be concluded that about 25% of quercetin was lost due to the photolytic reaction induced by UV exposure. Therefore, taking that into account, a screening effect of the microfibrous structure can exist, at certain amounts. Since the hydroxyl group on the C ring is known to be the main determinant for the photostability of quercetin [47], the drug located onto the surface can easily be degraded by UV-rays. Concerning the amount encapsulated inside the electrospun fibers, it is usually suggested that intermolecular hydrogen bonds could exist and have an effect on the protection of quercetin against UV irradiation. Therefore, energy of radiation is partially used for breaking the hydrogen bonds reducing [48]. The UV-Vis spectra of quercetin are reported in the right side as function of irradiation times. The released amount data along irradiation times have been modeled by setting a power law model as Q = Q_max_(1 + t_irr_)^b^ where Q_max_ is the maximum amount at t = 0 while the parameter b, being negative, confirms the decrease in Q released along the irradiation time (t_irr_).

### 3.6. Effects of Quercetin-Loaded Membrane on Cell Viability

**Figure 10:**
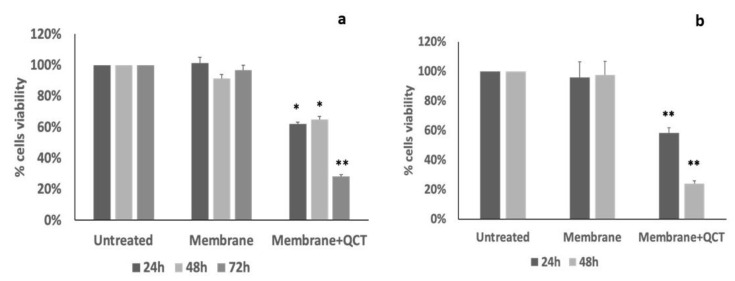
Cell viability assay. (**a**) MTT assay on KG1 cells after 24 h, 48 h and 72 h of treatment with membranes loaded with quercetin (direct contact); as negative control, not loaded membranes were used. (**b**) MTT assay on MRC5 cells after 24 h and 48 h of treatment with the conditioned medium, i.e., medium incubated for 24 h with membranes loaded with quercetin; as negative control, medium conditioned with non-loaded membrane was used. Data are reported are means ± standard errors of three independent experiments, each in triplicate and are expressed as the percentage of residual cell viability relative to the viability of untreated cells; * *p* < 0.05 and ** *p* < 0.01 versus untreated cells.

To evaluate if membrane-encapsulated quercetin retained the biology activity, we tested quercetin cytotoxic effects by performing an MTT assay. KG1 cells were grown in the presence of membranes loaded with quercetin and membrane alone. As shown in Figure 10a, membrane alone did not affect KG1 cell viability; instead, cells grown in the presence of membrane loaded with quercetin showed a significant reduction in cell viability after 24 h of treatment. The effect was maintained even after longer incubations (48 h and 72 h), with about 20% of residual cell viability after 72 h of incubation. Therefore, quercetin loaded on membranes is in an active form and the cytotoxic effect can be seen for the entire duration of the treatment.

To confirm that the observed effect on cells viability was dependent on quercetin released into the medium, we performed a MTT assay on MRC5 cells treated with the conditioned cell medium. As expected, the conditioned medium with membrane alone did not affect cell viability, whereas the conditioned medium with quercetin-loaded membrane reduced cell viability of about 40% after 24 h and of about 80% after 48 h of treatment (Figure 10b), indicating that released quercetin remained active for a prolonged time. In MRC5 cells, we also verified that quercetin added to the cell medium at a final concentration of 0.5 μM for 24 h reduced cell viability of 70%. Our next studies will focus on characterizing specific applications of membranes loaded with quercetin or other natural compounds with well-known anti-inflammatory, anti-microbic and anti-oxidant properties in the context of healing processes, in particular, wound repair. Different approaches in vitro have been already used to realize promising scaffolds for biomedical uses to improve drug delivery and healing action. Quercetin supply antibacterial property at osteogenic potential of magnesium-doped calcium silicate [49], important to avoid antibiotic administration and for successful bone regeneration. Nanofiber with nitric oxide-based treatment promotes wound healing by improve collagen deposition, cells growth and regulate inflammatory factors [50], while electrospun radially-oriented nanofiber mats together with herbal compound loaded hydrogel accelerated in full-thickness skin wound healing in diabetic disease [51]. Thus, in this prospective study, our research could be considered a preliminary proof of concept whose findings indicated the formation of a highly stable system with promising features for health applications.

## 4. Conclusions

Electrospun membranes of poly (ε-caprolactone) (PCL) and polyvinylpyrrolidone (PVP) were loaded with a fixed amount of quercetin as tunable drug delivery systems for health applications. The morphology and physical properties were studied and compared with neat PCL-PVP membrane. Morphological analysis showed for all the samples the formation of randomly oriented, defect-free cylindrical fibers. The loaded polymeric membrane showed a good homogenous distribution with average fiber diameter lower for unloaded membrane, probably due to a partial increase in conductivity due to the quercetin presence. The stability of quercetin in PCL/PVP systems was even proved by computational analysis. The retention degree was analyzed by testing four different solvents while the contact angle analysis was carried out by testing PBS, acidic and basic media. All membranes showed contact angles higher than 90°, indicating that the micro/nano fiber morphology is the main responsible for the strong reduction in the wettability of these materials. The release kinetic of quercetin was investigated in PBS and pH = 3 solutions which could simulate the skin lesion environment. The data were modeled through Gompertz model proving the slower release of quercetin in acidic medium, as demonstrated by the diffusion coefficients. The photostability of quercetin-loaded membrane was tested up to 10 h of irradiation with an UV-A lamp proving the stability of the encapsulated amount in the fibers even at high treatment times. Finally, the quercetin released from membranes preserves its biological activity as demonstrated by cytotoxic effects on cultured cells.

## Figures and Tables

**Table 1:** Parameters of kinetic models.

Model	PBS (pH = 7.4)		HCl SLN (pH = 3)	
		Values		Values
Zeroth	K_0_	0.66	K_0_	0.56
	R^2^	<0	R^2^	<0
Gompertz				
	α	0.83	α	0.97
	β	−2.7	β	−1.5
	R^2^	0.99	R^2^	0.98
Higuchi				
	K_H_	9.49	K_H_	7.93
	R^2^	<0	R^2^	<0
Korsmeyer–Peppas				
	K_R_	48.60	K_R_	37.41
	n	0.13	n	0.14
	R^2^	0.58	R^2^	0.73

## Data Availability

Not applicable.
